# Supplementary material for: Are carbon emissions trading and green financial instruments synergistic? -Comprehensive quantitative research based on content analysis
Source: PLoS One. 2024 Mar 7;19(3):e0298601. doi: 10.1371/journal.pone.0298601 (PMC10919601; doi:10.1371/journal.pone.0298601)
Supplement: S1 Data — (ZIP) [file pone.0298601.s001.zip › Data/地级市Z_F工作报告.zip!/╜¡╦╒/╤∩╓▌╩╨/└┤╫╘╣1⁄2╓┌║┼í╛┬φ┐╦╩2╛▌═°í┐_2011.docx]

各位代表：

现在，我代表扬州市人民政府向大会作工作报告，请予审议，并请市政协各位委员和其他列席人员提出意见。

2010年和“十一五”时期工作回顾

2010年，我们在中共扬州市委的正确领导下，团结和依靠全市人民，以科学发展观为统领，保增长、调结构、抓创新、惠民生，较好地完成了市六届人大三次会议确定的各项任务。预计全市实现地区生产总值2200亿元，增长13.6%；财政总收入400.88亿元，其中一般预算收入167.78亿元，分别增长29.3%和31%；城市居民人均可支配收入21750元，农民人均纯收入9295元，均增长12%。全市经济社会呈现持续稳定健康发展的良好态势。

一、经济持续较快发展

工业经济快速发展。全市规模以上工业实现产值5873亿元、增加值1389亿元、利税620亿元，分别增长34.7%、16.1%和40%。汽车船舶、机械装备、石油化工三大支柱产业产值4217亿元，增长38.4%。工业重大项目建设取得重要突破，上海大众整车、天威太阳能电池组件、华电天然气发电等一批重大项目开工建设。全市新增产值过百亿元企业1家、过50亿元企业6家。全市建筑业总产值1560亿元，增长20%。

现代农业持续发展。粮食总产287万吨，连续7年实现丰收。高效农（渔）业园区面积77.8万亩，新增27万亩；设施农（渔）业面积43.6万亩，新增17.5万亩。以海峡两岸（扬州）农业合作试验区为载体打造农业亮点工程，新引进农业项目634个。90家市级以上农业龙头企业销售收入增长20%。农民专业合作组织新增631个。通过复垦与综合整治新增耕地2.1万亩。开工建设万顷良田建设试点工程8.6万亩。水利、农业开发、粮食、供销、农机、气象、农业科研等工作进一步加强。

服务业加速发展。实现增加值814亿元，增长13.8%。出台鼓励发展软件和信息服务业专项政策，惠普外包、税友软件等一批项目落户。江苏（扬州）信息服务产业基地快速发展，扬州“智谷”、维扬动漫创意产业基地、邗江文化创意产业园等加快建设。扬州入选“中国城市信息化50强”。港口、石化、公铁水、商贸四大物流园区营业收入增长90.2%。成功举办第45届全国“旅交会”。接待来扬游客2700万人次，旅游业总收入300亿元，分别增长16.6%和18.6%。蜀冈－瘦西湖风景区晋升为国家5A级景区，东关街跻身“全国十大历史文化名街”。“富春”和“玉缘”商标成为中国驰名商标。冶春茶社台北分店开张营业。扬州农村商业银行、南京银行扬州分行挂牌营业，新建农村小额贷款公司7家。全市金融机构年末存款余额2431亿元、贷款余额1486亿元，分别增长17.6%和22.5%。

二、转型发展迈出新的步伐

产业结构进一步优化。“三新”产业产值571亿元，增长49.6%，占规模以上工业产值比重9.7%。服务业增加值占地区生产总值比重比上年提高0.9个百分点，软件和信息服务、现代物流、金融等生产性服务业增加值占服务业比重31%。高效农业、渔业面积分别占耕地和养殖水面的38.2%和70.2%。

科技创新成效显著。全市实现高新技术产业产值2224亿元，增长43.5%。全社会研发投入占地区生产总值比重1.9%。专利申请、专利授权分别增长34%和43%。我市“数字化测土配方施肥技术”获全国地级市唯一的国家农业科技成果推广一等奖。新设立省级以上“两站两中心”94家，新认定国家高新技术企业64家。成功举办“院士专家扬州行”活动和上海、武汉、成都“科技创新·产业合作”推介会，签订产学研合作协议286项。扬州成为国家“中小企业知识产权战略推进工程”首批实施城市、国家火炬计划智能电网特色产业基地。扬州光电科技创新园被评为全省首批创新型园区。江苏（扬州）数控机床研究院列入全省首批4家产业技术研究院建设计划。全市获省高层次创新创业人才项目26个。

内需拉动作用明显。全市社会消费品零售总额718亿元，增长18.6%。全市十大百货商场销售额、五大食品批发市场成交额分别增长23.3%和26.8%。全社会固定资产投资1332亿元，增长25.2%。市级重点固定资产投资项目累计开工303个。全市房地产投资165亿元，增长27.5%，商品房合同成交面积562万平方米。

节能减排扎实有效。全市完成节能减排年度目标。实施节能改造项目130个、循环经济项目36个、资源综合利用项目107个。对112家企业组织开展清洁生产审核。淘汰落后用能设备2371台（套），关闭“五小”企业91家。实施减排项目86个。扬州被选为国家可再生能源建筑应用示范城市。市经济技术开发区创成国家生态工业示范园区。

三、城乡统筹协调推进

重大基础设施加快建设。苏中江都机场开工建设。江海高速扬州段建成通车，安大公路全线贯通，京杭运河扬州段“三改二”工程全线竣工，南水北调东线一期工程三阳河、潼河、宝应站全面建成。沪陕高速江六段、扬州港5号泊位、宁启铁路复线及电气化改造、乌塔沟分洪道等工程抓紧建设。淮河入江水道治理工程、扬州西500千伏输变电工程获国家批复。

中心城市功能继续完善。编制完成扬州市城市总体规划（2010–2020）和14项专项规划。开工建设文昌路东延及跨廖家沟、芒稻河大桥和328国道连接线。新建和改建北环路、鉴真路等17条道路。加快建设广陵新城、临港新城、蜀冈生态新区和蒋王片区等。新城西区功能进一步完善。建成城北客运站并投入使用。新增公交车400辆、公交停车场和首末站6个。建成六圩污水处理厂二期和赵庄垃圾焚烧发电厂一期工程。宋夹城遗址保护展示工程、南门遗址展示馆建成开放。打通古运河至二道河、瘦西湖水上游览线。完善数字化城管体系。大力整治城市出入口环境。市区新增绿化面积150万平方米。

城镇化建设有力推进。着力构建中心城市、县城、重点中心镇、一般镇协调发展的城镇体系。县城建设步伐加快。研究制定了推进城镇化建设实施意见，出台了加快11个重点中心镇建设的奖励政策和考评办法。完成了全市村庄布局规划。新建农民新社区240个。深入推进国家生态市和国家森林城市创建工作。68个乡镇建成垃圾中转站，61个乡镇建成污水处理设施，51个乡镇通过国家生态乡镇考核或命名，创成生态村824个。江都、邗江国家生态县（市）创建通过省考核。全市新增造林面积16万亩，成片造林率居全省第一，森林覆盖率20.1%。

农村实事工程有效落实。新建改造农村公路280公里、公路危桥150座，所有行政村通上四级路。完成县乡河道、河塘疏浚3070万方。新改建无害化卫生户厕8万座、“一池三改”户用沼气池10100座。机插秧和秸秆机械化还田取得新的进展。新创成农村环境综合整治和“四位一体”长效管护达标乡镇30个。新创成全面小康村100个、新农村建设示范村31个。第三轮农村扶贫任务全面完成。

四、改革开放活力显著增强

招商引资取得新进展。成功举办“烟花三月”国际经贸旅游节和世界运河名城博览会。全市注册外资实际到账25.7亿美元，增长13.5%。新增民营企业注册资本金634亿元，增长16.7%。全市出口60亿美元，增长50%。完成外经营业额2.35亿美元，增长16%。新办境外投资项目21个。

园区实力明显提升。“八区二园”业务总收入5230亿元，增长40%。30强乡镇工业集中区实现产值2400亿元，增长40%。开发园区注册外资实际到账、地方一般预算收入、自营出口均占全市50%以上；基础设施建设新增投入198亿元，增长95%。

各项改革深化推进。完成市、县（市、区）政府和乡镇机构改革。全面完成集体林权制度改革。潍柴与亚星集团实现战略重组。出台《扬州市创新型经济金融助推计划》。汇银家电、长青股份成功上市。启动医药卫生体制改革，制定实施公立医院改革试点方案。推进文化体制改革，组建扬州报业传媒集团、广电传媒集团和市歌舞剧院公司。

五、社会建设富有成效

民生工程全面完成。城镇新增就业8.8万人，零就业家庭连续40个月保持动态清零，城镇登记失业率2.7%。新的市人力资源市场投入使用。全市企业职工基本养老保险净增缴费4.4万人，新农保参保率及基础养老金发放率均达99.7%。为全市19万企业退休人员上调养老金10%。全市城镇居民医保覆盖率95%，新型农村合作医疗覆盖率99.8%。新建残疾人康复中心2个、县级残疾人托养机构4个。建成市救助中心。住房公积金新增扩面6.88万人。市区新增经济适用房1260套、廉租房230套、公租房1850套（间），公有住房解危6.2万平方米，改造老小区17个、“城中村”15个，新建和改造农贸市场10个。建成市区第五水厂一期工程。区域供水市区和仪征实现全覆盖，高邮、江都、宝应按序时推进。加强物价监管，全年物价上涨3.4%，市区累计向低保对象、特困职工等发放物价补贴1700多万元。

社会事业全面发展。大力开展全国文明城市创建工作，城市公共文明指数和未成年人思想道德建设测评均列全省第一，“扬州好人”蔚然成风。启动义务教育优质均衡示范区建设，市区树人学校和育才小学跨区办学。邗江、广陵创成省教育现代化建设先进单位。全市本二以上万人上线率达30.4，居全省第一方阵。所有乡镇、街道建成文化站。组织文化惠民活动1万多场。建成包括市图书馆新馆、美术馆、音乐厅在内的市文化艺术中心。建成扬州工艺美术馆新馆、扬州工艺坊和中国淮扬菜博物馆。中国雕版印刷技艺入选联合国“人类非物质文化遗产名录”。基本建成“15分钟健康服务圈”，为城乡居民免费提供22项基本公共卫生服务。体育公园游泳跳水馆建成并运行。鉴真马拉松赛成为国际田联“银标”赛事，并被评为“全国首批体育旅游精品项目”。全市新增有线电视用户4万户。组织开展全国第六次人口普查。计划生育民生牵手工程和数字计生工作获省创新奖。市档案馆晋升为国家一级综合档案馆。成立扬州市社会科学院。成功举办日本东大寺鉴真坐像回扬省亲活动。台湾事务、侨务、外事和工会、青年、妇女、儿童、慈善、老龄等事业取得新的进步。

社会建设和管理有力有效。深入开展和谐社区创建活动，积极构建“四位一体”社区管理服务体制机制，推进社区工作站和社区服务中心建设，实现省级村民自治模范县（市）全覆盖。扎实推进“平安扬州”建设，公众安全感满意率和基本满意率连续8年保持在96%以上。建立健全矛盾纠纷排查化解机制，人民来信来访工作进一步加强。出台并实施加强校园安全20条措施，强化安全生产监管，加强食品药品安全专项整治，全市未发生有影响的重大案件和责任事故。积极做好国防后备力量建设和双拥工作，我市被南京军区表彰为“国防动员工作先进单位”。民防、仲裁、地震工作扎实开展。

六、服务型政府建设进一步加强

监督制度不断健全。自觉接受市人大及其常委会的法律监督、工作监督，主动接受市政协的民主监督，认真听取各民主党派、工商联、无党派人士和各人民团体的意见。办理人大议案1件、代表建议344件，办理政协提案488件。加强监察和审计监督。高度重视新闻舆论和人民群众监督，市政府公开电话和“寄语市长”回复率均在90%以上。

行政权力规范运行。出台《扬州市依法行政考核办法》，落实行政执法责任制，行政执法自由裁量权进一步规范。完成“五五”普法任务。加强政府新闻发布工作。建成行政权力网上公开透明运行系统，基本实现县级以上行政机关、行政权力事项和网上行政监察全覆盖。

能力作风有新的提升。制定并实施政府能力作风建设20条意见。试点推行并联审批，推进“三集中、三到位”，市级行政审批事项88%进入行政办事服务中心集中办理，对外承诺事项提前办结率97.1%。加强公务员学习培训，举办“新知学堂”讲座9期。聘请11位政府经济顾问。认真落实党风廉政建设责任制，查处了一批违法违纪案件。

在肯定成绩的同时，我们也清醒地认识到，全市经济社会发展中还存在一些深层次的矛盾和问题。主要是：经济实力和核心竞争力需要不断增强，转变发展方式还有大量工作要做；城乡二元矛盾依然突出，统筹发展任务十分艰巨；城市功能品质仍需提升，生态环境和城市管理有待继续改善；事关群众切身利益的就业、住房、教育、医疗、社会保障和增加收入等还有许多问题需要解决；政府及公务员队伍能力作风建设、反腐倡廉工作还要不断加强。这些问题需要我们高度重视，并切实采取措施认真加以解决。

各位代表！2010年工作任务的完成，标志着“十一五”规划确定的主要目标和各项任务胜利实现。五年来，在中共扬州市委的坚强领导下，全市坚持科学发展、克难求进、开拓创新，特别是克服了全球金融危机带来的巨大冲击和影响，经济社会保持了又好又快的发展。

这五年，是扬州综合实力不断增强、发展后劲大提升的五年。五年全市累计地区生产总值8185亿元，是“十五”的2.4倍，可比价年均增长14.3%；财政一般预算收入549.4亿元，是“十五”的3.4倍，年均增长27.6%；全社会固定资产投资4597亿元，是“十五”的3.5倍，年均增长26.6%。工业的主体地位进一步凸显，工业发展的脉络进一步清晰，工业经济的稳定性和成长性进一步增强。全市规模以上工业企业数、总产值、10亿元以上企业数，分别是“十五”末的1.9倍、4.1倍和7.5倍。

这五年，是扬州产业结构不断优化、创新型经济大突破的五年。全市三次产业结构由2005年的9.7：55.3：35调整为2010年的7.2：55.8：37。高新技术产业迅速壮大，新兴产业快速成长，现代服务业充满生机。先后创成汽车及零部件、数控金属板材加工设备、半导体照明、绿色新能源、智能电网等五大国家级特色产业基地，建成国家光电产业检测重点实验室、中科院扬州应用技术研发与产业化中心等一批重要研发平台。

这五年，是扬州各项改革不断深化、开发开放大推进的五年。行政体制改革不断深化。医药卫生、文化等体制改革逐步深入。五年累计注册外资实际到账84.6亿美元、出口202亿美元，分别是“十五”的3.9倍和3.5倍；新增民资注册资本金1579亿元，是“十五”的4倍。扬州经济开发区晋升为国家级经济技术开发区，扬州化工园区、邗江开发区、维扬开发区升格为省级开发区。

这五年，是扬州城乡统筹不断推进、名城建设大跨越的五年。“一体两翼”交通框架加快构建，“江河海沟通、公铁水联运”的现代交通格局基本形成。率先推进市区1100平方公里城乡统筹发展，全市城镇化率达54.4%。新农村建设成效显著，农村“新五件实事”和新农村建设“十大工程”持续推进，农村生产生活条件和农村环境明显改善。大力实施城市建设和环境提升工程，古城保护与新区建设同步推进，市区城市建设五年累计投入490亿元，是“十五”的1.6倍。扬州在全省首家荣获“联合国人居奖”。城市的知名度、美誉度进一步提高。

这五年，是扬州社会发展不断进步、人民生活大改善的五年。市委、市政府每年都出台和实施改善民生的“1号文件”。全市就业形势稳中向好，社会保障体系逐步健全、保障水平明显提高。基本公共服务及均等化取得新进展，一批公益设施建成开放。全市财政五年累计用于社会事业的投入超过前20年的总和。城乡居民收入年均分别增长13.8%和12.3%。人民群众幸福感明显提升。

各位代表！“十一五”时期我市经济社会发展所取得的成就，是全市上下认真贯彻党中央、国务院和省委、省政府正确决策的结果，是全市人民在中共扬州市委的坚强领导下开拓创新、踏实奋斗、团结拼搏的结果。在此，我谨代表扬州市人民政府，向全市人民，向人大代表、政协委员，向驻扬单位、各垂直管理部门和所有关心、支持、参与扬州建设和发展的社会各界人士，致以崇高的敬意和衷心的感谢！

“十二五”时期的目标任务

各位代表，根据《中共扬州市委关于制定扬州市国民经济和社会发展第十二个五年规划的建议》，市政府制定了“十二五”规划纲要（草案），提请本次大会审议。纲要（草案）提出了我市“十二五”时期经济社会发展的指导思想、发展目标和主要任务，经审议批准、颁布实施后，必将推动我市在新的起点上开创科学发展新局面。

“十二五”时期全市经济和社会发展的指导思想是：高举中国特色社会主义伟大旗帜，以邓小平理论和“三个代表”重要思想为指导，深入贯彻落实科学发展观，始终坚持发展第一要务，以坚持科学发展、建设“创新扬州、精致扬州、幸福扬州”为主题，以加快转变经济发展方式、“发展创新型经济、建设创新型城市”为主线，大力实施创新发展、城镇化、民生幸福、改革开放、可持续发展五大战略，更加注重发展的质量和效益、更加注重产业结构的优化和升级、更加注重城镇化水平的提升、更加注重区域统筹协调发展、更加注重人的全面发展、更加注重扬州“人文、生态、精致、宜居”特色的彰显，到“十二五”期末全面建成更高水平的小康社会并向基本现代化迈进，初步建成“古代文化与现代文明交相辉映的名城”。

今后五年全市经济社会发展的主要预期目标为：地区生产总值年均增长12%左右，财政一般预算收入年均增长20%，城市居民人均可支配收入和农民人均纯收入年均增长12%，节能减排完成国家和省下达目标任务。全面建成更高水平小康社会，为2020年基本实现现代化打下坚实基础。

实现“十二五”发展目标，必须牢牢把握主题和主线。突出坚持科学发展、建设“创新扬州、精致扬州、幸福扬州”这一主题，坚持发展第一要务，坚持以人为本，更加注重全面协调可持续发展，更加注重保障和改善民生。把加快转变经济发展方式、“发展创新型经济、建设创新型城市”作为“十二五”时期发展的主线，坚定不移地推进结构调整和自主创新，着力推动经济发展方式由主要依靠物质资源消耗向创新驱动转变、粗放式增长向集约型发展转变、城乡二元结构向城乡一体化发展转变。

实现“十二五”发展目标，必须深入实施五大战略。大力实施创新发展战略，坚持把创新发展作为经济社会发展的主引擎，努力把扬州建成创新产业发达、创新技术领先、创新载体优良、创新人才富集、创新体系健全的创新型城市。大力实施城镇化战略，坚持工业化、城镇化“双轮驱动”，加快形成城乡经济社会发展一体化新格局。大力实施民生幸福战略，不断提升保障和改善民生水平，促进人的全面发展和社会全面进步。大力实施改革开放战略，深入推进各项改革，加快经济国际化步伐。大力实施可持续发展战略，建设资源节约型、环境友好型社会。

实现“十二五”发展目标，必须认真落实六项重点任务。一是推进创新型城市建设。突出创新驱动，坚持教育和人才优先发展，期末全社会研发投入占地区生产总值比重达到2.5%，高新技术产业产值占规模以上工业产值40%以上。二是推进经济转型升级。推动先进制造业提升发展、现代服务业加速发展、现代农业规模发展。工业着力打造3个3000亿元级支柱产业、4个1000亿元级新兴产业和6个1000亿元级特色产业基地或集群。服务业着力构建“一城一市四中心”的现代服务业发展格局。农业着力建设一批重点现代农业园区和农业科技示范园区。三是推进城乡一体化进程。科学构建区域城镇体系，促进城乡基本公共服务均等化，期末城镇化率达62%。以扬州建城2500周年为时间节点，加快推进“精致扬州”建设，城市品质和核心竞争力进一步提升。进一步加快县域经济发展步伐。四是推进民生幸福建设。坚持以人为本、民生优先，打造充分就业城市，不断提升社会保障水平，加强社会建设和管理，创建全国文明城市，建设更加符合人民群众期盼、社会更加和谐安宁的幸福扬州。五是推进改革扩大开放。深度融入长三角，推进宁镇扬同城化。加快重点领域和关键环节改革步伐，着力构建充满活力、富有效率、更加开放、有利于科学发展的体制机制。打造开放型经济发展新优势，提升经济国际化水平。六是推进生态文明建设。坚持环保优先、节约优先方针，创成国家森林城市和国家生态市，全面提升城乡生态文明水平。

各位代表！实现“十二五”发展目标，是一项光荣而又艰巨的任务。我们要同心同德、攻坚克难、拼博奋斗，确保“十二五”发展的宏伟蓝图在扬州大地上变为美好的现实。

2011年主要工作任务

今年是中国共产党建党90周年，是“十二五”开局之年，是建设“三个扬州”的关键之年。全市经济社会发展的主要预期目标为：地区生产总值增长12%。财政总收入增长20%，其中一般预算收入增长22%。全社会固定资产投资增长25%。研发投入占地区生产总值比重2%以上。社会消费品零售总额增长14%。城市居民人均可支配收入和农民人均纯收入增长12%。居民消费价格总水平涨幅控制在4%以内。城镇登记失业率控制在4%以内。节能减排完成省下达任务。

完成上述目标，实现“十二五”良好开局，必须全力以赴促增长、转方式、提品质、控物价、惠民生。着力抓好八项工作：

一、突出创新转型，加快提升工业发展层次和水平

狠抓工业重大项目建设。集中资源，重点抓好上海大众整车、天威太阳能电池组件、华电天然气发电、康博6000吨高纯硅和潍柴亚星新能源汽车等重大项目建设。继续推进晶澳扬州基地、中海运江都基地、中外运仪征基地、玛切嘉利等重大项目的增量扩产。加快远东100万吨精对苯二甲酸、建滔200万吨重油催化裂解等投资过亿美元在谈项目的落实。全市工业投资1400亿元，新开工10亿元以上项目40个。

提高自主创新能力。加强创新载体建设，重点推进市经济技术开发区新兴产业研发园、邗江大学科技城、广陵信息产业基地、维扬文化创意产业园、仪征汽车科技城、江都软件园、高邮光电科技园、宝应输变电装备科技园等载体建设，新建省级以上“两站两中心”50家以上。突出企业创新主体地位，推动企业技术需求与高等院校、科研院所科技成果的深度对接，促进扬州大学及地方高校的办学和科研与扬州经济社会发展相融合，新签产学研合作协议250项，建成一批产学研合作战略联盟。抓好企业品牌创建工作。加快引进国内知名理工类院校、科研机构落户扬州。认真实施“绿扬金凤计划”。全年新认定国家高新技术企业50家以上，获批省级以上高新技术产品和重点新产品400项，实现高新技术产业产值2500亿元。

做大做强新兴产业。围绕打造“中国光电城”完善产业链，着力发展以晶澳、天威为龙头的国家绿色新能源产业，积极发展半导体照明及背光源终端应用产品。大力发展高压超高压电缆、智能电网终端设备、应用软件等，争创国家智能电网示范试点城市。推进电子书与文化、出版、教育、网络融合发展。全市“三新一网一书”产业产值增长30%以上。继续跟踪研究和布局其它新兴产业的发展。

拓展提升支柱产业。积极开发生产新能源汽车、环保型发动机，加快进入海工装备制造领域。依托现有优势，促进机械装备业实现智能化、柔性化、成套化。以乙烯、丙烯、芳烃以及氯碱类产品为主体，促进石化产业链增粗接长。全市汽车船舶、机械装备、石油化工产业分别实现产值960亿元、1900亿元和1200亿元。加快运用信息技术改造、提升传统产业，培育“两化融合”试点示范企业100家。全市实现建筑业产值1800亿元，增长15%。

促进资源节约、环境友好。积极发展节能型产业，创建节能示范企业22家。加快电机系统等10项先进节能型技术的应用，做好可再生能源建筑应用示范市建设工作，推动全社会节能。大力发展循环经济，组织开展80家企业清洁生产审核，实施30项循环经济试点项目。支持扬州环保科技产业园发展。

二、推进城乡统筹，大力发展县域经济和农村经济

加快发展现代农业。加大新品种、新技术推广力度。进一步提升发展农业亮点工程，打造高效设施农业特色乡镇和专业村。壮大发展农业龙头企业，提升农民专业合作，推进“农超对接”和农业产业化。加大海峡两岸（扬州）农业合作试验区项目引进力度，农业新增利用外资3亿美元、民资和工商资本70亿元。新增高效农（渔）业面积和农业适度规模经营面积各20万亩。继续大力推广水稻机插秧和秸杆机械化还田及综合利用。

加强农村基础设施建设。大力推进农田水利建设，疏浚县乡河道、整治村庄河塘2500万方，除险加固小水库24座。加快中低产田改造，推进15万亩高标准农田建设。新建农村公路200公里，改造公路危桥100座。新改建无害化卫生户厕6万座、“一池三改”户用沼气池7600座。实施新一轮农村电网改造。

大力推进城镇化建设。深化市区城乡统筹发展规划。继续加强县城建设。编制完成11个重点中心镇镇区建设用地控制性详规，优先实施20项基础设施和民生项目。加大户籍制度改革力度。有序、有力推进土地股份合作、万顷良田建设工程试点和城乡建设用地增减挂钩。

推进县域经济争先进位。围绕实现县域经济“三年跨越、争先进位、创新发展”计划目标，继续大力实施工业强县战略，做大做强主导产业，加快发展新兴产业。全力发展省级开发区和重点乡镇工业集中区，着力打造专业特色园区和产业特色乡镇。

三、聚焦重点行业，提速发展现代服务业

强力发展软件和信息服务业。进一步明确全市软件和信息服务业主攻方向，围绕呼叫中心、数据中心、容灾备份中心和嵌入式、信息安全、物联网等软件，组织对国内重点企业和培训机构点对点招商。开拓境外服务外包市场。全面推进中心城区各区和江都等有条件县（市）软件和信息服务业载体建设，全市新增载体面积55万平方米。推动传统产业信息化改造和社会信息化应用，推进市政府部门网站整合和集中数据中心建设。

大力发展旅游业。深化文化旅游综合开发，启动扬州古城和古运河风光带国家5A级景区创建工作。加快“一县一品”和市县旅游资源整合、联动发展步伐。加大城市旅游推介，突出对北京、上海等大城市和日韩、欧美等国家地区的市场开拓。办好大陆和台湾2011江苏（扬州）灯会。继续壮大发展瘦西湖旅游发展集团有限公司。优化旅游发展环境，强化餐饮、宾馆、旅行社等行业管理，加大星级宾馆尤其是五星级宾馆建设力度。全年接待旅游总人数、旅游总收入均增长20%以上。

加快发展现代物流业。提升完善四大物流园区综合配套服务能力。推进扬州港与太仓港深度合作。加快出口加工区保税物流仓储、长江石化物流中心、医药物流配送基地、化工园区公共液体码头等项目建设。推进制造业企业主辅分离和社会物流资源整合。引进培育一批第三方、第四方物流品牌企业。

加速发展文化产业。重点发展文化创意、影视制作、出版印刷、演艺娱乐、文化会展、动漫等行业。加快推进邗江文化创意产业园、市工艺美术集聚区等载体建设。积极培育文化龙头企业和骨干企业。

创新发展金融服务业。健全投融资体系，新增上市公司2家以上。大力发展信托投资、金融租赁、融资担保、投资银行等金融产品和专营机构，新引进2家银行机构，建成1家村镇银行，完成11个重点中心镇小额贷款公司布点。

积极发展其他服务业。提升发展传统商贸业，稳步发展连锁经营、电子商务等新型业态。提升优化文昌商圈，继续打造京华城全生活广场等城市新型商贸集聚区。推进农村“新网”工程和“家电下乡”。培育壮大家庭服务业。大力发展农业服务业。

四、深化改革开放，不断增强发展活力

积极推动宁镇扬同城化。推动建立多层次多形式的宁镇扬区域协商协调机制。推动宁镇扬同城化战略规划不断细化。推动宁镇扬三市规划、环保、公共服务等一体化。加快推进城际轨道交通、直通高速公路建设，打造宁镇扬半小时交通圈。

进一步提高开放开发水平。继续办好“烟花三月”国际经贸旅游节。大力实施产业链招商，精心组织战略性新兴产业招商活动，全市注册外资实际到账增长20%。制定完善综合奖励政策，大力发展总部经济。调整优化出口产品结构，全市出口增长20%。深入开展以“创新转型”为主题的园区“二次创业”活动。

壮大发展民营经济。强化政策扶持，优化发展服务，进一步推动自主创业。积极引导和推动民营企业向现代企业制度转变。更大力度地组织开展民资招商活动，全市新增民营企业注册资本金增长20%，总投资亿元以上项目100个，开工项目当年注册率90%以上。鼓励和支持行业商会发展。

深入推进各项改革。完善国有资产管理体制，推进企业战略性重组。继续深化医药卫生、文化体制改革。提高财政科学化、精细化管理水平，加强对已有重点支出项目绩效评价，对新增财政较大专项试行绩效预评估。积极推进其他领域改革。

五、放大精致特色，进一步加强中心城市建设

完善提升城市规划。按照新一轮城市总体规划，优化完善城市用地布局和道路交通规划，重点深化城市综合交通枢纽及轨道交通规划。抓好古运河沿线及重点地段的城市设计，编制历史文化名城保护规划。推进中心城区控制性详规全覆盖。完善规划设计方案优选制度，强化对城市规划的批后监管。

实施新一轮重大基础设施建设。加快苏中江都机场、沪陕高速江六段、宁启铁路复线及电气化改造、扬州港“一港三区”、淮河入江水道整治工程、扬州西500千伏输变电工程等项目建设。推动实施长江深水航道治理工程。推进连淮扬镇铁路和长江公铁大桥、京沪高速公路扩容及南延过江工程、省国信扬州电厂三期工程等重点项目的前期工作。

全面提升城市品质。建成328国道连接线，基本建成文昌路东延工程。开工建设瘦西湖地下通道和江都路延伸、开发路东延等工程。加快古运河沿线、文昌路等“五路一环一河”景观改造，整治甘泉路、广陵路和彩衣街等传统街巷。继续推进文化博览城建设。开工建设瘦西湖旅游文化广场、动物之窗二期工程。继续提升“双东”片区，建成街南书屋和东关美食文化广场，整修岭南会馆等历史建筑，修缮传统民居200户以上。加快建设城市规划展示馆和市民中心、运博会永久会址、科技馆、体育公园体育场等项目。启动实施扬州城国家遗址公园建设。加强城市防洪排涝工程建设。新建7座污水提升泵站。建设天然气利用二期工程。

加强城市精细管理。大力开展市容环境综合整治，健全城市管理长效机制。扩大市区数字化城管覆盖范围，进一步提高处置效率。市区街巷实现市场化保洁全覆盖。继续对公交站台、指示路牌等“城市家具”进行统一更新。建设市区小贩中心3个。市区新建垃圾中转站2座、公共厕所14座、公共停车场（库）4－6个。加强城市道路交通管理，加强学校周边环境整治。

六、坚持以人为本，努力提升民生幸福

促进就业再就业。围绕打造充分就业城市的目标，制定实施完善创业就业服务体系、保障重点行业和企业劳动力需求、促进重点人群就业、创业带动就业四项行动计划。完善城乡统筹就业政策，建设“15分钟就业服务圈”，重点做好高校毕业生、农村转移劳动力、城镇就业困难人员、退役军人等就业服务工作。全市新增城镇就业4.6万人、农民转移就业2万人以上。

加强物价监管调控。切实抓好新一轮“菜篮子”工程，市区启动实施“菜篮子1161计划”。加强粮油肉等基本生活品储备。强化价格监测预警，将价格监测从主要关注主副食品价格拓展到关注整体民生价格。大力开展价格专项检查，整顿市场价费秩序。做好普通商品住房价格备案工作。严格执行涉及保障群众基本生活稳定的各类收费标准和条件，落实对弱势群体价费优惠政策，适时实施低收入群体价格补贴。深化资源节约和生态环境价格改革。

完善提升社会保障。提高城乡低保和农村“五保”标准。全市城镇职工基本养老保险净增缴费3.2万人，城镇职工、城镇居民基本医疗保险和农村新型合作医疗参保率稳定在98%以上，工伤保险新增参保3.2万人。全市新农保参保率稳定在95%以上，基础养老金发放率稳定在98%以上。启动市区被征地农民和失地农民转换参加企业职工基本养老保险。

办好民生实事工程。启动实施居民收入六年倍增计划。加强食品安全监督。实施市区第一水厂水质提标改造。继续推进区域供水工作，江都、高邮实现全覆盖，宝应达80%以上。整治改造市区老小区15个、“城中村”15个，新建和改造农贸市场11个。优化调整公交线路，推进公交优先。加强保障性住房建设。市区新建经济适用房1000套、公租房2000套（间）、廉租房100套，实施公有住房解危6万平方米。全市住房公积金扩面6万人以上。加强老小区物业管理。大力发展慈善事业和老龄事业。全市免费对残疾人技能培训1500人以上，帮扶残疾人就业1000人以上。做好农村扶贫和集体经济薄弱村帮扶工作。

七、加快发展社会事业，提高社会建设和管理水平

坚持教育优先发展。贯彻落实国家和省中长期教育规划纲要，大力推进教育改革创新和教育现代化建设。切实加强学前教育，加大公办幼儿园建设力度，广陵、维扬各新建一所公办幼儿园。推动义务教育优质均衡发展，市区建成京华中学、维扬中学新校区、育才小学东部分校。优质特色发展高中教育，创新发展职业教育，优化发展高等教育。加快实施中小学校舍安全工程。确保来扬务工人员子女100%入学。

提高医疗卫生服务水平。全面推进公共卫生服务项目和基本药物制度实施。启动区域卫生信息平台建设。加强城市医院与社区卫生机构的对口支援和双向转诊，加快基层卫生人才的引进和培养，启动农村医疗急救网络建设。完成苏北医院三期病房楼、市人医东区医技楼、市中医院综合楼扩建工程建设，基本完成市二院异地重建。

繁荣发展文化事业。加强文化设施建设，完成市文化馆迁建并达到国家一级馆标准，建设200个农村文化广场。制定出台政策和办法，促进使用好、管理好市文化艺术中心。支持艺术团体和艺术家开展文艺创作。继续举办各类公益演出和“市民日”活动，办好市第七届少儿艺术节和农民艺术节。继续挖掘整理扬州历史文化，加强策划、包装和对外宣传。做好非物质文化遗产保护、展示和传承工作。

协调推进其他各项社会事业。继续稳定低生育水平，提高人口素质。大力开展全民健身活动，推进“三边”工程建设，举办好鉴真国际半程马拉松赛。维护妇女儿童合法权益，深入推动妇女创业和发展。加快档案馆新馆建设。继续做好二轮修志工作。理顺红十字会管理体制，加强救助能力建设。做好民族宗教工作。做好新疆新源县等对口支援工作。加强国防后备力量建设，争创新一届全国“双拥模范城”。

创新加强社会管理。认真实施“六五”普法。加快构建“四位一体”社区治理模式，强化社区管理和服务。积极发展社会组织，壮大志愿者队伍。加强社会稳定风险评估，畅通和规范群众诉求表达、利益协调、权益保障渠道。继续深化“平安扬州”建设，完善社会治安防控体系。进一步健全大调解机制，强化社会矛盾调处中心建设，做好群众信访工作。促进仲裁事业和法律援助事业健康发展。加强应急管理体系建设，提升突发公共事件预防和应对处置能力。继续抓好安全生产、消防、食品药品质量监管。

八、咬定创建目标，扎实推进“三创一申”工作

认真落实2011年创建各项任务和责任，坚持创建为民、创建惠民、创建靠民，扎实推进“三创一申”工作。狠抓全国文明城市创建工作。对照全国文明城市测评体系要求，硬件抓达标，软件抓提升，基础抓夯实，广泛开展文明单位（行业）、文明村镇、文明社区等群众性精神文明创建活动，落实长效管理措施，切实加强未成年人思想道德建设，力争创成全国文明城市。狠抓国家森林城市创建工作。实施5大类18项重点绿化工程，全市新增造林15.5万亩，市区新增绿地100万平方米以上，力争创成国家森林城市。加快国家生态市创建步伐。完善县乡污水收集主干管网系统，提高污水收集率和处理率。全市80%的乡镇创成国家级生态镇，所有县（市、区）通过国家生态县考核。做好申遗工作。细化“扬州盐商历史遗迹”申遗各项基础工作，发挥大运河联合申遗牵头城市作用。办好世界运河名城博览会。

努力建设人民满意的政府

各位代表，实现“十二五”和今年的目标任务，使命光荣、责任重大。新的形势、新的任务要求我们必须加快转变政府职能、创新优化政府服务，努力建设对人民负责、为人民办事、受人民监督、让人民满意的政府。

着力建设服务型政府。始终坚持群众立场，牢记为人民服务的宗旨。自觉、主动深入群众，倾听群众呼声，高度关注并认真解决事关群众切身利益的热点难点问题。精细分析各类经济主体需求，既为所有经济主体的发展营造机会均等、公平竞争的市场环境，又为重点行业、重点企业提供专业化的精准服务。主动帮助基层和企业向上争取政策支持和资金扶持。重视来扬客商和旅行旅游者的需求，参照国际通行城市管理方式和服务标准，加强城市标识、问询、紧急援助等服务设施建设，提供及时、周到、便捷的帮助和服务。

着力建设法治政府。自觉接受人大法律监督和工作监督、政协民主监督，主动听取民主党派、工商联、无党派人士和社会各界意见。认真办理人大代表议案、建议和政协提案。深度推进政务公开。完善重大事项集体决策、专家咨询论证、新闻发布会制度。继续办好市政府公开电话、“寄语市长”和部门服务热线。加强行政复议规范化建设。严格执行行政监察法、公务员法和党政领导干部问责暂行规定。强化审计监督。

着力建设创新型政府。把开拓创新作为政府工作的鲜明导向。面对发展过程中出现的新情况和新问题，注重对国内外促进经济发展和加强城市管理的经验、做法的研究和借鉴，注重对已有成功探索的总结和推广，注重从工作失误中吸取教训，开阔眼界、拓宽思路，创新办法、破解难题。以人民群众的服务需求为中心，加强政府工作业务流程重组和部门协同，注重发挥大众传媒和新型服务载体作用，提高政府各项惠民政策的通达性，实现为民服务简便、快捷、高效。充分利用社会力量，整合社会资源，增强政府公共服务能力。继续举办“新知学堂”，加强公务员队伍新知识、新业务、新技能的学习培训。

着力建设廉洁政府。深入开展廉政建设和反腐败斗争，认真落实党风廉政建设责任制，进一步建立健全教育、制度、监督并重的惩治和预防腐败体系。强化公务员队伍理想信念和廉洁自律教育。加强对公共服务部门的职责监管，开展对部门重要岗位的满意度测评。坚持为民、务实、清廉，坚决抵制和反对一切消极腐败现象，坚决纠正损害群众利益的不正之风，以干净干事、坦诚坦荡、清正廉洁的政府形象取信于民。

各位代表！我们已经踏上“十二五”新的征程。宏伟目标激励我们团结拼搏，美好蓝图需要我们同心绘就。让我们高举中国特色社会主义伟大旗帜，以邓小平理论和“三个代表”重要思想为指导，深入贯彻落实科学发展观，在中共扬州市委的领导下，解放思想，开拓创新，锐意进取，扎实工作，为完成2011年和“十二五”时期的各项目标任务，为建设“创新扬州、精致扬州、幸福扬州”而努力奋斗！
